# A Novel DNA Vaccine Technology Conveying Protection against a Lethal Herpes Simplex Viral Challenge in Mice

**DOI:** 10.1371/journal.pone.0076407

**Published:** 2013-10-03

**Authors:** Julie L. Dutton, Bo Li, Wai-Ping Woo, Joshua O. Marshak, Yan Xu, Meei-li Huang, Lichun Dong, Ian H. Frazer, David M. Koelle

**Affiliations:** 1 Coridon Pty Ltd, Brisbane, Queensland, Australia; 2 Department of Medicine, University of Washington, Seattle, Washington, United States of America; 3 Department of Laboratory Medicine, University of Washington, Seattle, Washington, United States of America; 4 Vaccine and Infectious Diseases Division, Fred Hutchinson Cancer Research Institute, Seattle, Washington, United States of America; 5 Diamantina Institute, University of Queensland, Brisbane, Queensland, Australia; 6 Department of Global Health, University of Washington, Seattle, Washington, United States of America; 7 Benaroya Research Institute, Seattle, Washington, United States of America; McMaster University, Canada

## Abstract

While there are a number of licensed veterinary DNA vaccines, to date, none have been licensed for use in humans. Here, we demonstrate that a novel technology designed to enhance the immunogenicity of DNA vaccines protects against lethal herpes simplex virus 2 (HSV-2) challenge in a murine model. Polynucleotides were modified by use of a codon optimization algorithm designed to enhance immune responses, and the addition of an ubiquitin-encoding sequence to target the antigen to the proteasome for processing and to enhance cytotoxic T cell responses. We show that a mixture of these codon-optimized ubiquitinated and non-ubiquitinated constructs encoding the same viral envelope protein, glycoprotein D, induced both B and T cell responses, and could protect against lethal viral challenge and reduce ganglionic latency. The optimized vaccines, subcloned into a vector suitable for use in humans, also provided a high level of protection against the establishment of ganglionic latency, an important correlate of HSV reactivation and candidate endpoint for vaccines to proceed to clinical trials.

## Introduction

Polynucleotide vaccines offer potential advantages over licensed formats, including heat stability, relatively low cost, and the ability to encode multiple antigens. While there are licensed veterinary polynucleotide vaccines/therapeutics for melanoma in dogs, West Nile virus in horses, infectious hematopoietic necrosis in salmon, and for porcine reproductive and respiratory syndrome [1]–[4], DNA vaccines have demonstrated suboptimal immunogenicity in human studies. In this paper, we utilized technology designed to enhance DNA vaccine immunogenicity to engineer a codon-optimized and ubiquitinated herpes simplex virus 2 (HSV-2) DNA vaccine based on the envelope protein glycoprotein D (gD2), and evaluated its performance in a murine HSV-2 viral challenge model.

More than 500 million people world-wide are infected with HSV-2 [5], the main causative agent of genital herpes, a disease which causes not just discomfort but considerable psychological distress. HSV-2 infection is believed to facilitate the transmission of HIV-1 [6], [7] and if acquired in late pregnancy can result in neonatal encephalitis. While some vaccine candidates have been able to induce neutralizing antibody and CD4 T cell responses they have been unsuccessful in protecting against HSV-2 infection and it is now recognized that an effective vaccine will most likely require a balanced CD4+ and CD8+ response (reviewed in [8], [9]).

Correctly synthesized and folded viral structural proteins present conformational epitopes that induce virus-specific neutralizing antibodies. However, they have evolved to resist proteolytic degradation, which is a prerequisite for efficient presentation to the immune system to induce helper and cytotoxic T cell responses [10]–[12]. Inclusion of a single ubiquitin sequence at the N-terminus of a protein has been shown to enhance proteasome-dependent degradation, resulting in an increased cellular immune response *in vivo* [13]. Ubiquitin-fused DNA vaccines induce enhanced cellular responses targeted against papillomavirus [14], melanoma [15], porcine reproductive and respiratory syndrome virus [3], and *Trypanosoma cruzi* [16]. Mutations to disrupt protein structure have also been used to enhance vaccine-induced T cell responses [17]. Use of a mixture of non-ubiquitinated and ubiquitinated antigen-encoding constructs should in theory induce both antibody and CD8 responses, as the translated ubiquitinated protein will be rapidly degraded to peptides suitable for processing to the Class I presentation pathway, whereas the translated non-ubiquitinated protein will retain structure and therefore present appropriate B cell epitopes, and be taken up by endolysosomes for Class II presentation. We have previously demonstrated that combining expression plasmids encoding non-ubiquitinated and ubiquitinated human papillomavirus L1-E7 proteins in a polynucleotide vaccine could induce enhanced L1 neutralizing antibodies and E7-specific CD8 responses [14]. Recently, a study of Wilms’ Tumor DNA vaccines showed that use of a mix of ubiquitinated and non-ubiquitinated antigen-encoding constructs was superior to immunization with either construct alone [18]. The current study examined the efficacy of a similar immunization strategy for an HSV-2 vaccine. We evaluated the immunogenicity of codon-optimized, ubiquitinated and/or non-ubiquitinated gD2-encoding constructs in mice and examined their ability to protect against lethal infection and to reduce HSV-2 DNA copy number in the vagina and dorsal root ganglia (DRG). Results from standard DNA vaccine vectors were extended and confirmed in vectors suitable for human administration.

## Results and Discussion

To investigate the effect of simultaneously encoding ubiquitinated and non-ubiquitinated protein on efficacy of a gD2 polynucleotide vaccine, various polynucleotide expression constructs were prepared for gD2. The gene sequence encoding HSV-2 gD was codon-modified according to an algorithm designed to improve antibody responses, and sub-cloned into pcDNA3 (Invitrogen, Carlsbad, CA) to make a plasmid designated pcDNA3-O-gD2 (D). The insert in this construct was used to make a truncated version of the codon-modified gD2 gene, lacking the signal peptide- and transmembrane domain-encoding sequences, inserted immediately downstream of, and in-frame with, a codon-optimized sequence encoding a single ubiquitin polypeptide to make pcDNA3-O-Ubi-gD2_25–331_ (UD). A full-length gD construct was also made with de-optimized codons and designated pcDNA3-W-gD2 (W).

### Immunization with Mixed Codon-optimized Ubiquitinated and Non-ubiquitinated gD2 Constructs Provides Superior Protection Against Lethal HSV-2 Challenge than Ubiquitinated gD2 Construct Alone

**Figure 1:**
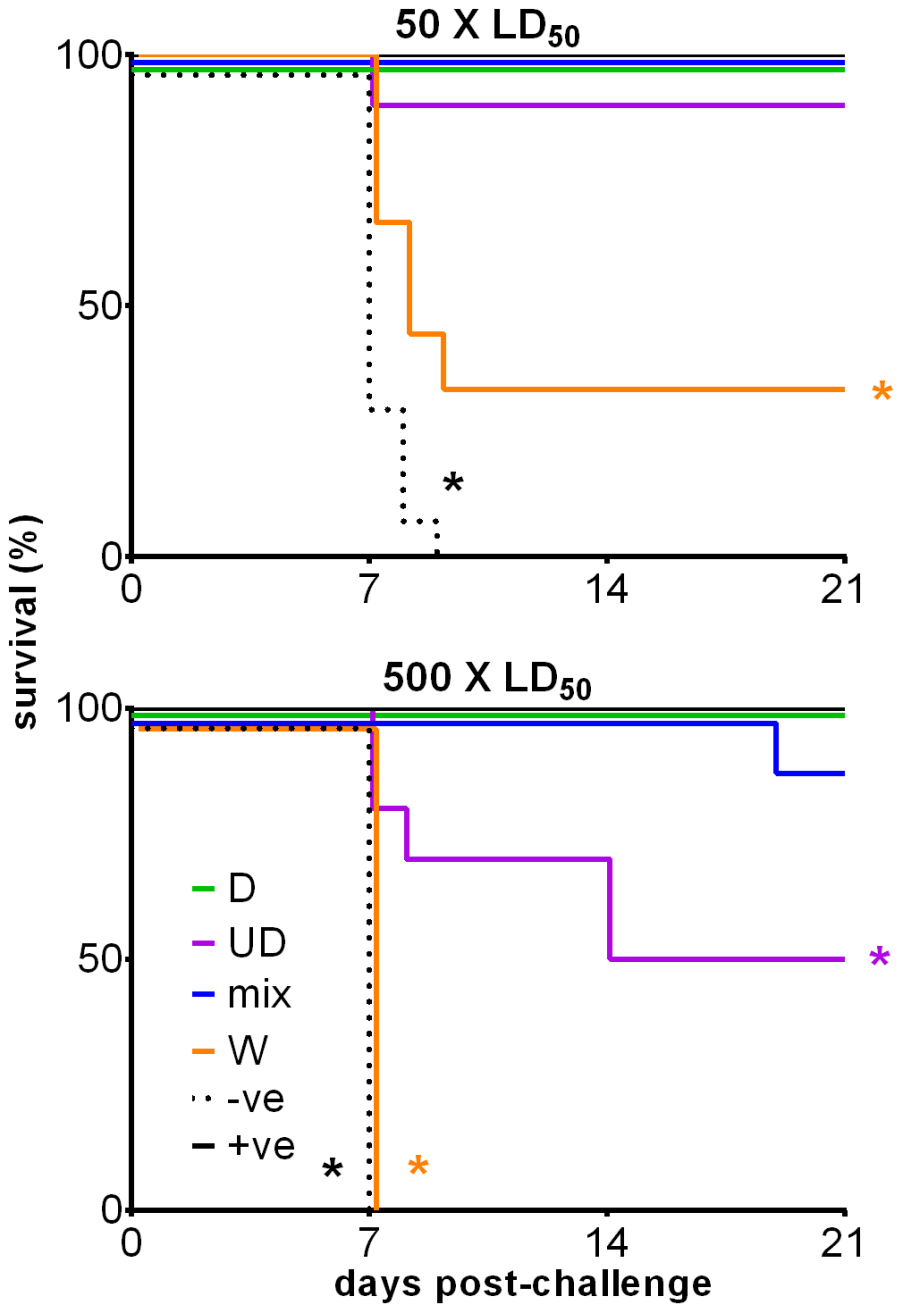
Survival, following HSV-2 challenge, of mice immunized with codon-optimized polynucleotides encoding antigen and ubiquitinated antigen. Mice sensitized with progesterone were challenged intra-vaginally with 50 (1.55×10^4^ p.f.u.) or 500×LD_50_ of live HSV-2 strain 186, after three immunizations with 20 µg of plasmids expressing HSV-2 gD and/or ubiquitin-tagged truncated gD, and survival monitored. 10 mice/group/challenge dose were used (with the exception of the positive control which used 5 mice/challenge dose). Survival of mice immunized with a plasmid containing a codon-optimized insert encoding full length HSV-2 gD pcDNA3-O-gD2 [D], a plasmid encoding a ubiquitin-tagged truncated HSV-2 gD pcDNA3-O-Ubi-gD2_25–331_ [UD], a mixture of D and UD [mix], a plasmid containing a codon de-optimized insert encoding full-length gD pcDNA3-W-gD2 [W], mouse thymidine kinase-deficient live HSV-2 strain 333 (5×10^5^ p.f.u./mouse) [+ve], or empty pcDNA3 vector [-ve] is shown. For the 500× challenge, the survival differences between UD and D and between UD and mix were significant as determined by a log-rank (Mantel-Cox) test. The survival of W and -ve was significantly different from the vaccine and +ve control groups at both challenge levels. * P<0.05. This data has been published in patent US 2011/0287039 A1.

The D and UD plasmid constructs, individually or mixed at a 1∶1 ratio, were tested for protective efficacy against HSV-2 challenge in BALB/c mice (Fig. 1). Mice were intradermally immunized with 20 µg of plasmid DNA three times at two week intervals, sensitized with progesterone, and challenged with HSV-2 two weeks after the final immunization. Mice immunized with each of the optimized vaccines (D, UD and the mixture) were protected against a 50× LD50 dose. Against a 500× LD50 challenge, the UD vaccine alone conferred significantly less protection than either the D or the mixture vaccines (P<0.05 by the log-rank (Mantel-Cox) test). The non-optimal codon (W) construct gave little protection, with 30% and 0% of mice in this group surviving the 50× and 500× LD50 challenge doses, respectively.

**Figure 2:**
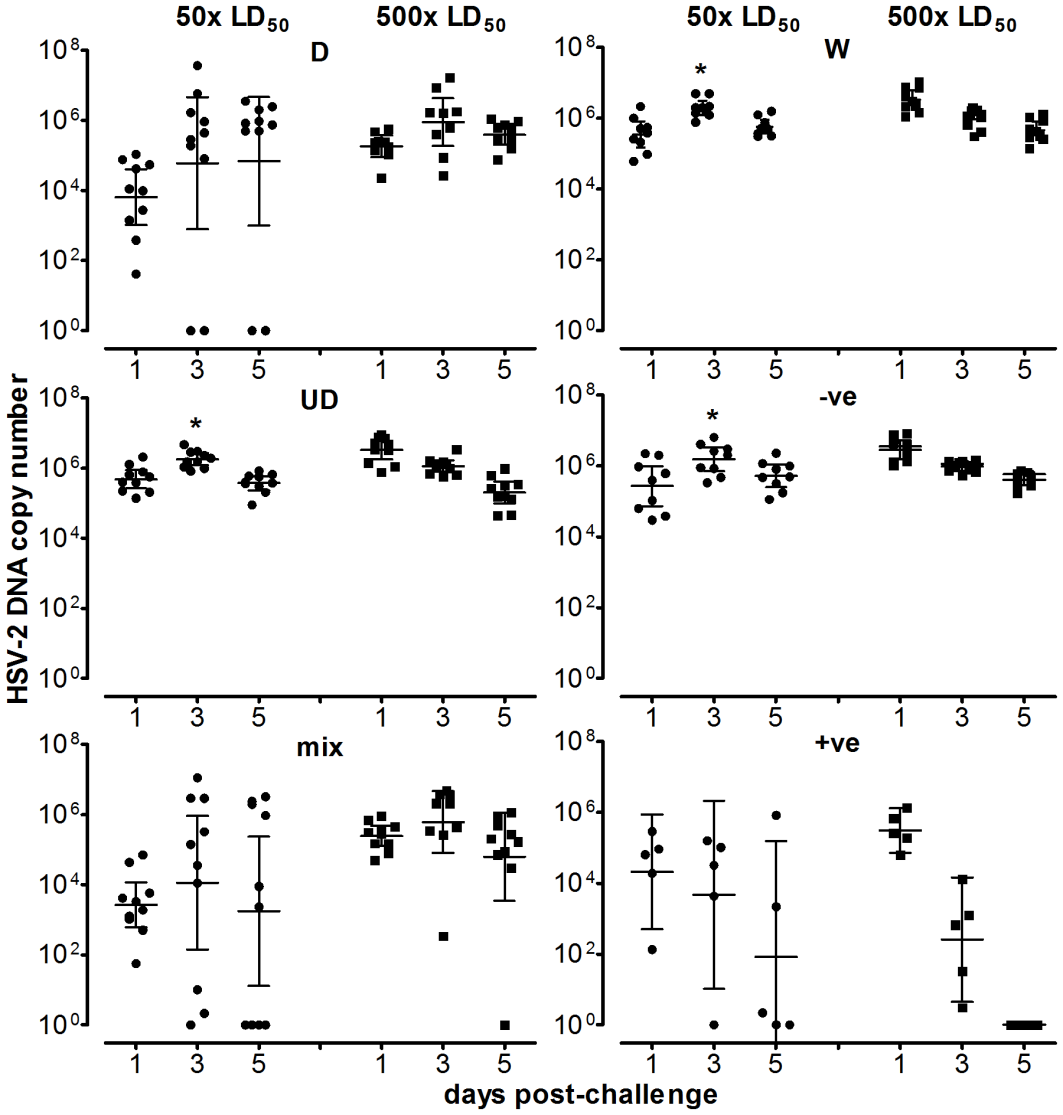
HSV-2 DNA copy number in vaginal swabs, from immunized mice, taken after HSV-2 challenge. The HSV-2 DNA copy numbers in vaginal swabs taken from mice 1, 3 and 5 days post-intravaginal challenge with 50 (1.55×10^4^ p.f.u.) or 500×LD_50_ of live HSV-2 strain 186 are shown. 10 mice/group/challenge dose were used (with the exception of the positive control which used 5 mice/challenge dose). The geometric means and 95% confidence intervals are indicated. The differences between the HSV-copy numbers over days 1 to 5 for the mixed vaccine versus UD alone, W and empty vector control were significant at the 50× challenge level (*P<0.05 by the Kruskal-Wallis test and Dunn’s post test analysis of the area under the log10 copy number-time curves). This data has been published in patent US 2011/0287039 A1.

Vaginal swabs, taken 5 days after viral challenge from mice immunized with the mixture vaccine or D alone, showed a reduction in viral DNA copy number to below the level of detection of the assay for 40% and 20% of mice, respectively, at the 50× LD50 challenge dose (Fig. 2). Recipients of the empty vector, W, or UD alone, each had high copy numbers of virus detectable at day 1, 3 and 5 (∼9×10^4^ to 2×10^6^ at day 5). In the 50× LD50 challenge groups, the virus copy number over days 1 to 5 in mice immunized with the mixed D and UD vaccine were significantly lower than in mice immunized with UD alone, W, or the empty vector control (P<0.05 by the Kruskal-Wallis test and Dunn’s post test analysis of the area under the log10 copy number-time curves). The positive control live attenuated HSV-2 vaccine provided superior vaginal load protection at both inoculum levels.

### The Mixed Vaccine Gives Rise to a Balanced T Cell and Antibody Response not Achievable with Either Construct Alone

**Figure 3:**
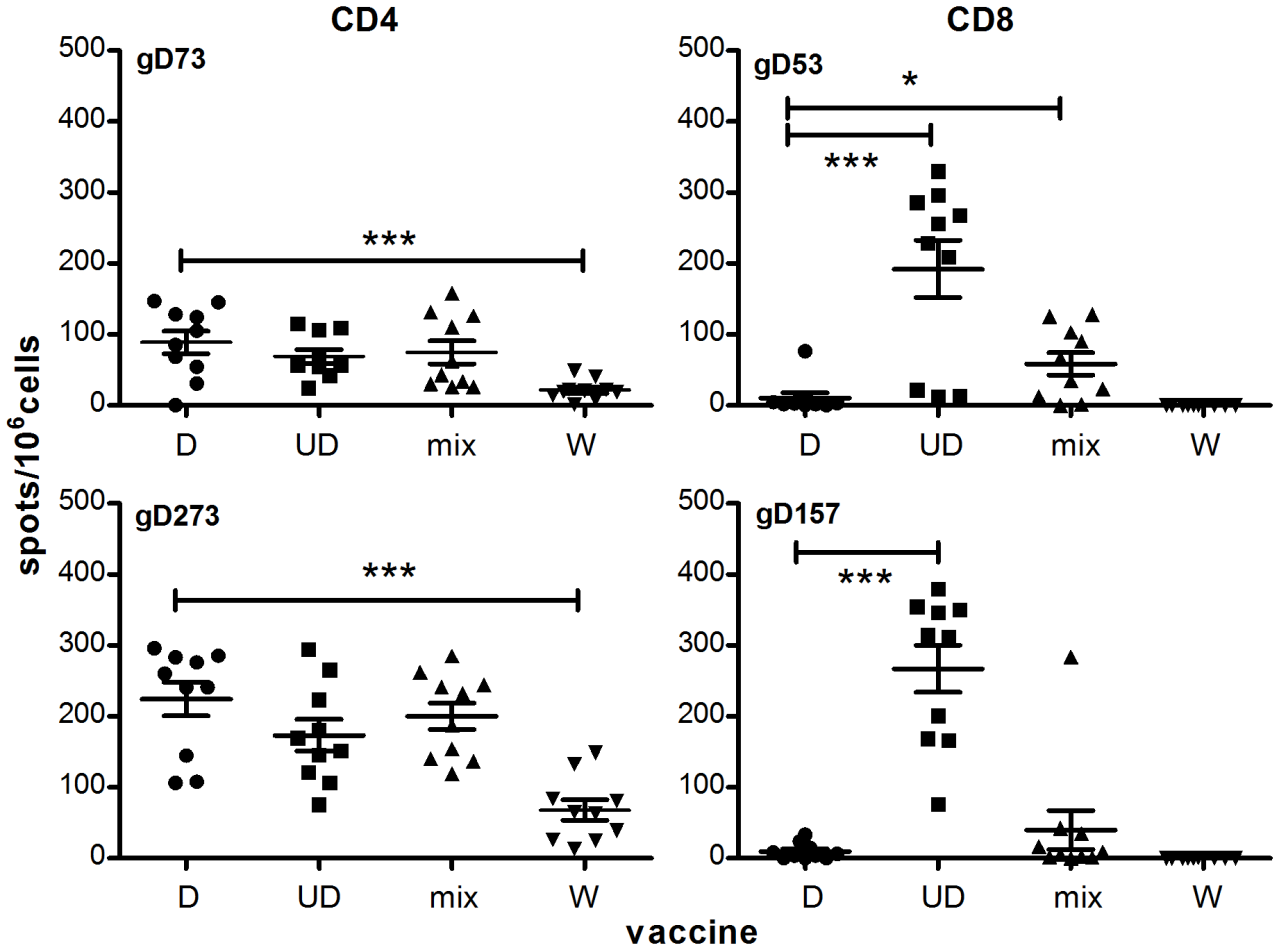
HSV-2 peptide-specific T cell responses induced in mice immunized with pcDNA3-based gD2 vaccines. 2 weeks after the final immunization, splenocytes were collected and the frequency of peptide-specific IFN-γ-secreting T cells assessed by ELISPOT. Peptides 73 and 273 include known gD2 CD4 epitopes and peptides 53 and 157 include gD2 CD8 epitopes. Each point represents triplicate assays on a single mouse. Responses in empty vector immunized animals were <5 s.f.u./10^6^ splenocytes (not shown). The average number of spots in the no peptide control wells for each vaccine was subtracted from the data prior to graphing. *** P<0.001, * P<0.05 by unpaired two-tailed t-test relative to spleen cells from mice immunized with D. Means ± SEM are indicated. The data in this figure is from 3 repeat ELISPOTs, the data from the first experiment has been published in patent US 2011/0287039 A1.

Cellular immune responses to the D and UD plasmid constructs, and to a 1∶1 mixture, were examined in immunized unchallenged animals. Spleen cells from immunized BALB/c mice were tested for interferon-γ (IFN-γ) secretion in response to known major histocompatibility complex-I and -II restricted peptide epitopes of gD2 in H-2^d^ mice [19] (Fig. 3). The D and UD vaccines and the D/UD mixture each elicited a response to CD4-restricted peptides gD73 and gD273. UD also elicited a strong response to CD8-restricted peptides gD53 and gD157, significantly greater than that elicited by D alone (P = 0.0003 and P<0.0001, respectively, by unpaired two-tailed t-test). The D/UD mix elicited a significantly greater response than D to CD8-restricted peptide gD53 (P = 0.0141), though not as large a response as UD (P = 0.006). The reason for the different CD8 responses between the D/UD mix and UD alone are unknown. As discussed in other reports of mixed DNA vaccination [20], competition for translation apparatus within cells or competition for antigen processing pathways, even between the closely related D and UD constructs, could be occurring. The mixed vaccine did have less of each plasmid, 10 micrograms, than the 20 micrograms given in the univalent vaccine. Responses to empty vector were <5 spot forming units (s.f.u.)/10^6^ splenocytes (not shown).

**Figure 4:**
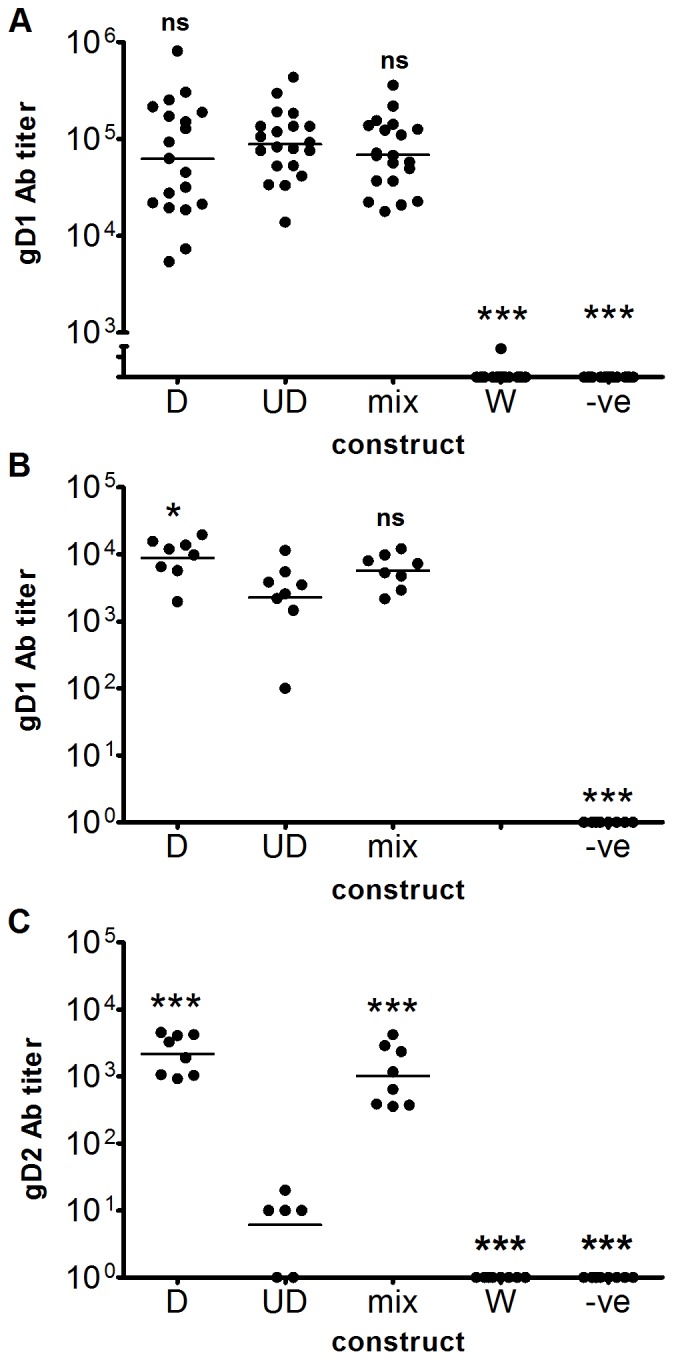
Antibody responses to codon-optimized polynucleotide vaccines encoding antigen and ubiquitinated antigen. A. Antibody titers of mice after three immunizations with 20 µg of plasmids expressing HSV-2 gD and prior to HSV-2 challenge, measured using yeast-expressed gD1 protein. B and C. Antibody titers of mice immunized as for A but not challenged, measured using yeast-expressed truncated gD1 protein and CHO cell-expressed truncated gD2 protein, respectively. Mice were immunized with pcDNA3-O-gD2 [D], a plasmid encoding a ubiquitin-tagged truncated HSV-2 gD (pcDNA3-O-Ubi-gD2_25–331_ [UD]), a mixture of the two plasmids [mix], a codon de-optimized construct pcDNA3-W-gD2 [W], or empty pcDNA3 vector [-ve]. For parts A-C, * P<0.05, *** P<0.001, ns = not significant as measured by one-way ANOVA followed by Tukey’s Multiple Comparison test. Significance is shown relative to the UD construct. Log titers were compared so that the distributions were approximately Gaussian. n = 20, 8 and 8 for parts a, b and c, respectively. The geometric means are shown. The data in parts A and C have been published in patent US 2011/0287039 A1.

Sera from the immunized mice, both challenged and unchallenged, were tested for antibody to a yeast-expressed gD1_21–339_ protein by enzyme-linked immunosorbent assay (ELISA; Fig. 4A and B, respectively). Sera from the unchallenged mice were also tested in an ELISA using a mammalian cell expressed C-terminally hexa His-tagged gD2_26–331_ (Fig. 4C). The reason for using two different ELISAs is historical: prior to collaboration the Coridon lab in Australia used their own in-house gD2 while the University of Washington lab in Seattle used a commercial source of gD1. At the time a commercial source of gD2 was not available and as gD1 and gD2 are 82% amino acid identical, determined by aligning full-length gD1 and gD2 from strain 17+ and HG52, respectively, in MegAlign V6 (DNASTAR Inc., Madison, WI) with 88% identity over residues 26–331, gD1 was used to estimate gD2 antibodies. In animals that were subsequently challenged (Fig. 4A) and in unchallenged animals (Fig. 4B), the D and UD constructs and the D/UD mixture induced strong gD-specific antibody responses, as assessed against the yeast-expressed gD1. The sera from the unchallenged mice was also tested against mammalian-expressed gD2, however while the D construct and mixture gave similar, high titers, the titers generated by the UD construct were much lower (Fig. 4C). While D encoded a full length gD protein, and the UD construct was N and C terminal truncated, the test antigens were both N and C terminal doubly truncated, and thus the observed differences were not due to detection of epitopes at either terminus of gD, but may be due to differences in glycosylation as the gD2 antigen, made in CHO cells, displays mammalian glycosylation patterns, while the gD1 antigen made in yeast likely has alternate glycosylation. The overall pattern of immune reactivity is consistent with the UD construct eliciting superior CD8 responses, but failing to generate antibodies to some epitopes present in native gD2, a hypothesis amenable to future research.

Constructs encoding ubiquitinated gD2 with or without the trans-membrane domain induced a strong CD8 response (data not shown) suggesting that ubiquitination, rather than removal of the transmembrane domain is likely to be responsible for the observed difference in immune response. In support of this, immunization with plasmid encoding ubiquitinated *Trypanosoma cruzi* antigen protected normal mice but did not protect mice deficient in components of the immunoproteasome [16]. As the signal-encoding sequence was removed from both non-ubiquitinated and ubiquitinated-antigen-encoding constructs in this study, the difference in immunogenicity of their constructs could be attributed to the ubiquitin. Additionally, we have previously shown [14] that addition of a ubiquitin sequence to an intracellular Human Papillomavirus L1-E7 fusion protein, with no membrane insertion sequence, significantly enhanced the CD8 response to this antigen. It therefore seems likely that the ubiquitin signal rather than the presence of a signal sequence led to the enhanced cellular response seen in the present study.

### The Number of Immunizations, Dose and Delivery Regime Affect Immune Responses, Protection Against Challenge and Viral Persistence in the DRG

In preparation for clinical trial of the polynucleotide vaccine, inserts from D and UD were sub-cloned into NTC8485 (Nature Technology Corporation, Lincoln, NE), a vector considered more suitable for use in humans than pcDNA3, to produce NTC8485-O-gD2 (nD) and NTC8485-O-Ubi-gD2_25–331_ (nUD). NTC8485 lacks an antibiotic resistance marker, contains the minimal sequences essential for bacterial replication of the plasmid and mammalian expression, and has modified eukaryotic mRNA leaders and terminators to minimize homology with the genome and therefore reduce the chance of chromosomal integration.

As immunization with a mixture of D and UD apparently gave similar efficacy but with a more balanced cellular response than immunization with D alone, and the CD8 response induced by the UD construct was diminished by co-delivery with the D construct, we next determined whether immune response and protective efficacy following immunization could be improved by altering the quantity of immunized plasmid, the ratio of nUD to nD plasmid, or the number of immunizations.

**Figure 5:**
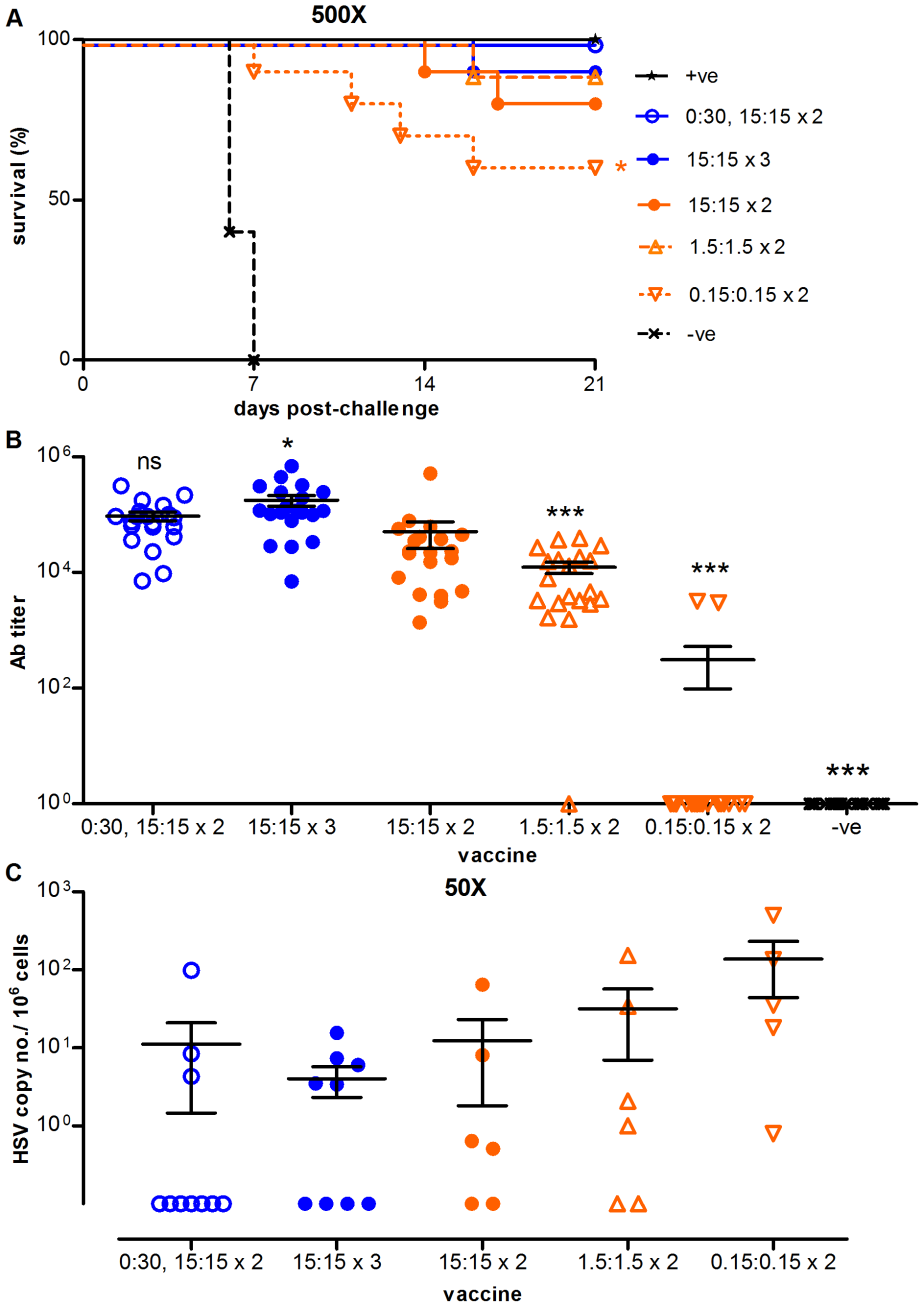
Comparison of different vaccination regimes using NTC8485-O-gD2 and NTC8485-O-Ubi-gD2_25–331_ in the HSV-2 challenge model. A. The effect of immunization on the survival of mice vaginally challenged with 500× LD_50_ (1.55×10^5^ p.f.u.) of live HSV-2 strain 186. +ve refers to the positive control mouse thymidine kinase-deficient live HSV-2 strain 333 (5×10^5^ p.f.u./mouse); -ve refers to empty NTC8485. The survival differences between the [0.15∶0.15×2] and [0∶30 then 15∶15×2] and between the [0.15∶0.15×2] and positive control group were significant as determined by a log-rank (Mantel-Cox) test (*P<0.05). Survival rates of the active vaccine groups were all significantly higher than the negative control (P<0.05). B. IgG antibody responses to HSV gD1 on the day of challenge (day 42). Sera from mice immunized with the gD vaccines or empty vector control were assayed by ELISA. One-way ANOVA followed by Tukey’s Multiple Comparison test was used to compare vaccines (*** P<0.001, * P<0.05, ns = not significant relative to the 15∶15×3 vaccine group) as this method allows comparison of three or more unmatched groups. Log titers were compared so that the distributions were approximately Gaussian. C. Dorsal root ganglia HSV-2 DNA copy number at euthanasia in survivors of the 50× LD_50_ challenge 62±2 days after challenge. Each dot represents pooled ganglia from a single animal. The ratios indicate the µg of non-ubiquitinated construct: µg of ubiquitinated construct; “x2” or “x3” indicate that the vaccine was administered twice or three times, respectively. The means with SEM are shown in B and C. Ten mice/group/challenge dose were used (with the exception of the positive control which used five mice/challenge dose).

The majority of mice challenged with 50× LD50 or 500× LD50 of HSV-2 survived, regardless of the dose of administered nD or nUD plasmid (Fig. S1A and Fig. 5A, respectively). Less effective protection was seen with the lowest dose of plasmid (0.3 µg), although the survival of 70% (50× LD50) and 60% (500× LD50) of mice immunized with this dose is noteworthy, as induced gD-specific antibody was measured in just 2 of 20 immunized mice (Fig. 5B). This dissociation between detectable serum antibody and survival has been previously noted in this model [18], and in fact, using live attenuated HSV-2 vaccines as in the positive control used in this study, genetic knockout and antibody depletion studies show that protection is mediated by T cells rather than antibodies [21], [22]. This illustrates the complexity of protective responses to HSV-2.

No relationship was apparent between the choice of plasmid backbone and the degree of protection, as a 1∶1 mixture of 15 µg of nD and nUD in the NTC8485 vector gave similar protection to that observed with 10 µg of D and UD in the pcDNA3 vector in the earlier experiment. Consistent with the first challenge study, mice immunized with empty vector died by day 8 after 50× and 500× LD50 viral challenge, whereas mice immunized with live attenuated HSV-2 survived (Fig. S1A and Fig. 5A).

**Figure 6:**
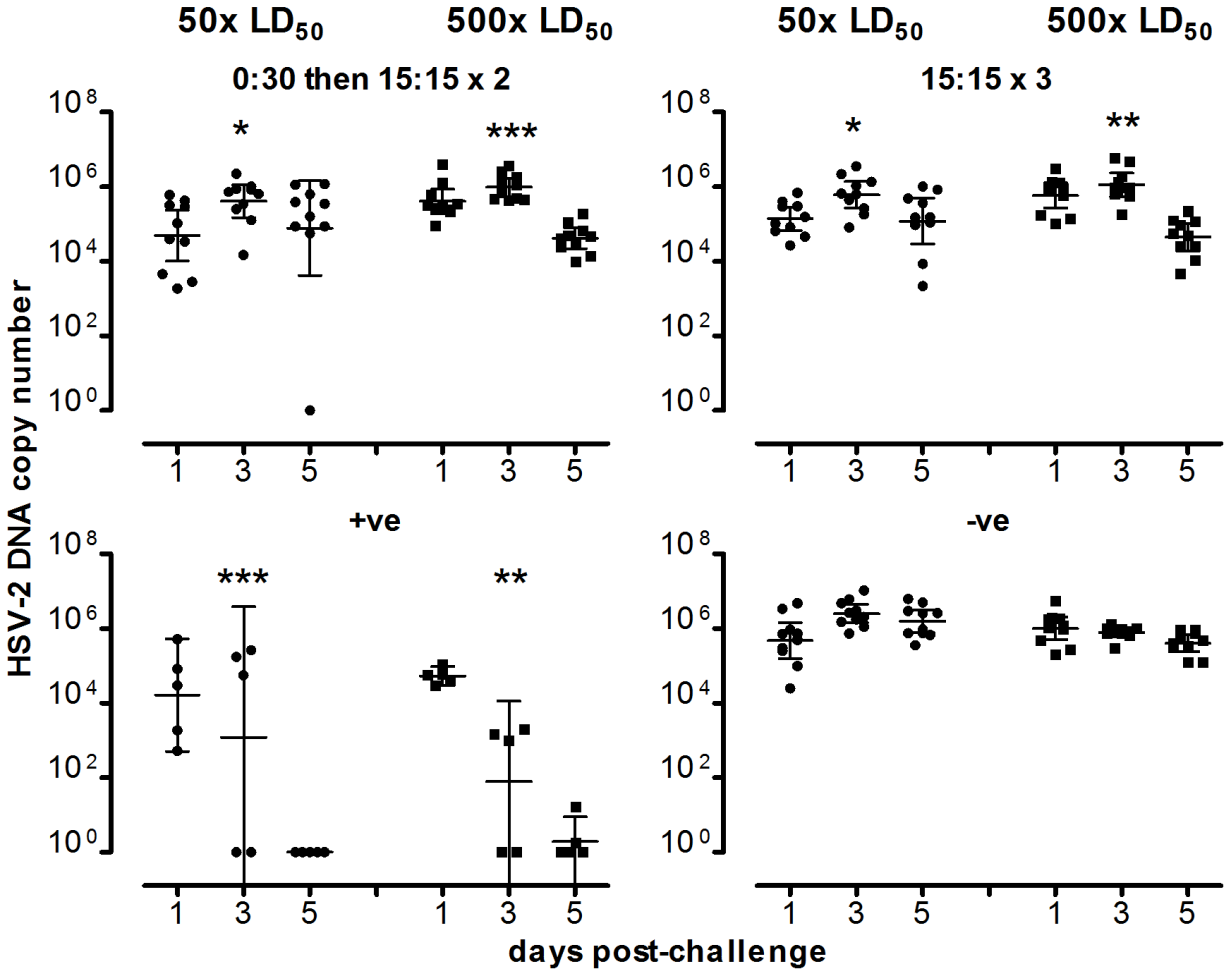
HSV-2 DNA copy number in swabs from mice immunized with NTC8485-based vaccines taken after challenge. Prior to intravaginal challenge with 50 (1.55×10^4^ p.f.u.) or 500×LD_50_ of live HSV-2 strain 186, mice were immunized with the indicated vaccines. 10 mice/group/challenge dose were used (with the exception of the positive control which used 5 mice/challenge dose). Swabs were taken 1, 3 and 5 days post-challenge. The ratios indicate the µg of non-ubiquitinated construct: µg of ubiquitinated construct; “x2” or “x3” indicate that the vaccine was administered twice or three times, respectively. +ve refers to the positive control mouse thymidine kinase-deficient live HSV-2 strain 333 (5×10^5^ p.f.u./mouse); -ve refers to empty NTC8485. The geometric means and 95% confidence intervals are shown. The vaginal swab HSV-2 copy numbers for the 15∶15×3 and the 0∶30, 15∶15×2 treatment groups (and the positive control) after challenge were all found to be significantly lower than the for the negative control group (*P<0.05, **P<0.01, ***P<0.001 by Kruskal-Wallis and Dunn’s multiple comparison test of area under the log10 copy number-day curves).

Mice were examined for virus persistence in the DRG 62±2 days after viral challenge. Of 10 mice immunized three times with a 1∶1 mix of 15 µg of nUD and nD, and challenged with 50× LD50 HSV-2, 9 mice survived to day 62±2 after challenge, and of these 4 were negative for HSV-2 in the DRG. For those mice primed with 30 µg of nUD and then given 2 immunizations with a 1∶1 mix of 15 µg of nUD and nD, 7 out of 10 challenged with 50× LD50 and 3 of 10 mice challenged with 500× LD50 were clear of virus in the DRG at day 62±2 (Fig. 5C and Fig. S1B). Lesser doses of mixtures of nUD and nD also gave significant protection against lethal challenge, and against persistence of virus in the DRG. Because the thymidine kinase-deficient HSV strains also established ganglionic latency in mice [23], we were not able to determine with our current PCR test if any of the test vaccines were superior to this positive control vaccine regarding ganglionic protection. HSV-2 DNA was detected in vaginal swabs taken 1, 3 and 5 days after challenge (Fig. 6 and Fig. S2) for both the 50× and 500× LD50 challenge groups. However the vaginal HSV-2 load over days 1 to 5 in mice immunized with the 30 µg of nUD followed by 2×1∶1 mix, and in mice immunized three times with 30 ug of the 1∶1 mix, was in each case significantly lower than in mice immunized with the empty vector control (P<0.05 by the Kruskal-Wallis test and Dunn’s post test analysis of the area under the log10 copy number-time curves).

### General Discussion

We obtained 100% protection with a codon-optimized, non-ubiquitinated gD2 construct. Absent a wild-type control, we cannot definitively isolate the effect of codon optimization in the absence of ubiquitin. We do note reports of similar models using wild-type gD2 sequences had poor protection with doses of up to 100 micrograms of gD2 DNA [24], [25]. While no animal HSV-2 model mimics human pathogenesis, we used mice due to the excellent immunologic tools and the ability of HSV-2 to predictably establish ganglionic latency, a feature similar to humans. We were able to clearly show that ubiquitinated and mixed vaccines were biologically active in the ganglionic load readout. Animals or humans with protection of the ganglia by definition cannot reactivate. We also showed superiority for CD8 responses with the ubiquitinated and mixed vaccine. Elicitation of CD8 responses has been challenging for non-vectored or non-viral vaccine approaches that have the advantage of safety. CD4 responses are important to prime and maintain CD8 responses at the levels of dendritic cell priming and CD8 T cell transition to memory [26], [27] in murine HSV models, such that a coordinated response is desirable. Given the difficulties of animal HSV-2 models, ganglionic protection and CD8 responses represent incremental gains in surrogate endpoints worthy of further research.

Considerable challenges remain in the design of an effective preventative or therapeutic HSV vaccine. The GSK adjuvanted, truncated gD2 phase III clinical trial [28] indicates that a vaccine that elicits antibodies and a Th1 CD4 T cell response can partially prevent new HSV-1 but not HSV-2 infections. While HSV-1 endpoints were not protocol-mandated, these data and the human papillomavirus vaccine mechanism of action [29] suggest that antibodies can be useful for an epithelial sexually transmitted viral infection such as HSV-2. The structural details of HSV gD interactions with nectin-1, the primary epithelial receptor [30], and HVEM, a neuronal receptor [31], are coming to light such that optimized immunogen design may now be possible. Study of neutralizing antibodies to both HSV-1 and HSV-2 in human and animal experiments may help to elucidate if specific responses are associated with protection. The guinea pig model, which does provide an opportunity to study recurrent genital HSV-2, could also be studied in the future. The DNA approach used herein can freely manipulate immunogen sequence without the consequences for immunogen manufacture inherent in the subunit approach. Potent enhancement of both immune and protective responses to HSV-2 vaginal challenge in mice and guinea pigs using cationic lipids with gD2 DNA [32], [33] augment prior work with protein-coding genetic adjuvants [34], suggesting that strong, balanced immune responses can be achieved using DNA. Induction of local responses is also effective in mouse HSV models [35] and the ability of the regimens studied herein to induce local immunity is worthy of future study. Possibly, the lower protection of the ubiquitinated construct alone compared to the mixed vaccine, despite better CD8 and equal systemic CD4 and antibody responses could be due to differences in local effectors.

## Conclusion

It has previously been demonstrated in humans that an antibody response to gD2 equivalent to the levels seen in natural infection is insufficient to confer protection from HSV-2 infection, and it is thought that an effective vaccine against HSV-2 will require a balanced immune response. This is reflected in current approaches to HSV-2 vaccine design which aim to induce/enhance cellular responses and include for example the use of adjuvant-enhanced subunit DNA vaccines [32], [36], live-attenuated viral vaccines [37], [38], or CTL-epitope containing peptides, alone or mixed and complexed with heat shock protein 70 [39], [40]. In this paper, we have demonstrated that the relatively simple approach of mixing codon-optimized ubiquitinated and non-ubiquitinated gD2-encoding constructs produces a balanced cellular and humoral response which is able to confer high rates of survival in a mouse model of HSV-2 infection and to reduce latent viral DNA load in the DRG, suggesting the vaccine may be able to reduce viral latency and shedding. The best vaccination regime, as measured by the prevention of DRG latency, included immunization with the plasmid encoding ubiquitinated protein to prime the cellular response followed by two immunizations with a 1∶1 mixture of plasmids encoding ubiquitinated and non-ubiquitinated protein. Three immunizations with the 1∶1 mixture, a regime more likely to be adopted for a commercial vaccine, also performed well. These two vaccine regimens conferred survival against viral challenge, had statistically significantly lower vaginal HSV-2 copy numbers than the negative control mice after 50× challenge and resulted in very low or undetectable latent viral loads in the dorsal root ganglion. Recruitment is underway for a phase 1, proof-of-concept, open label, single escalating dose study to assess the safety, tolerability and immunogenicity of the 1∶1 mixed vaccine in humans (Australian Therapeutic Goods Administration #2013/0324).

## Materials and Methods

### Ethics Statement

The HSV-2 challenge experiments were approved by the University of Washington IACUC under protocol 4095-02 issued to David Koelle. The animal experiments not involving HSV-2 challenge were approved by the University of Queensland animal ethics committee, approval numbers AIBN/DI/184/09/DI/CORIDON and DI/506/09/UQ.

### Plasmids

gD2 expression constructs were made using pcDNA3 (Invitrogen, Carlsbad, CA) and inserts based on nucleotide sequence from strain HG52 (Genbank ID: Z86099.2) modified according to our codon preference table [41]. Inserts were synthesized with overlapping ∼35–50 mer oligonucleotides (Sigma-Aldrich, St Louis, MO) using the method of Smith et al [42] modified as follows: an initial denaturation step at 94°C for 30 s followed by 25 cycles of 94°C for 15 s, 55°C for 30 s and 68°C for 1 min, and a final step of 68°C for 3 mins. Inserts were ligated into the vector, sequenced, and errors fixed using the Quikchange II Site- or Multi Site-directed Mutagenesis kit (Stratagene, La Jolla, CA). The truncated optimized gD2_25–331_ part of UD was made by PCR from D. All constructs were propagated in *Escherichia coli* strain DH5α, and purified using a Nucleobond Maxi Kit (Machery-Nagal, Düren, Germany). The GenBank accession numbers for D, UD and W are JF304427, JF715063 and JF304429, respectively. Inserts were subcloned from pcDNA3 into NTC8485 to make nD and nUD and near-GMP grade plasmid DNA of these and control NTC8485 manufactured (Nature Technology Corporation, Lincoln, NE).

#### Mice

For antibody studies/IFN-γ enzyme-linked immunospot (ELISPOT) and viral challenge experiments, female BALB/c mice were obtained from Monash University (Melbourne, Australia) and from Jackson Laboratory (Bar Harbor, ME), respectively. Mice were housed under specific pathogen-free conditions and used at 6–8 weeks of age.

#### Immunization procedure

For antibody studies, 8 mice were used per group. Three independent ELISPOT experiments were carried out, using 4, 3 and 3 mice/group. For infection studies, 20 mice were used per group, with half challenged with 50× LD50 and half with 500× LD50 doses of HSV-2. Mice were immunized three times at 2 week intervals intradermally in the pinna of each ear with plasmid DNA. For the first challenge study and the ELISPOT and antibody studies, 20 ug of DNA (i.e.10 ug/ear) was delivered per immunization day. For the second challenge study, mice were immunized with phosphate-buffered saline (PBS) alone or 0.3–30 ug DNA for the first immunization and with 0.3–30 ug DNA for the subsequent two immunizations. Serum was collected by retro-orbital bleed on days 0, 28 and 42. Spleens were collected two weeks after the final immunization. For both challenge studies, each positive control mouse was intravaginally immunized with 5×10^5^ p.f.u. thymidine kinase-deficient live HSV-2 strain 333 after progesterone treatment.

### IFN-γ ELISPOT

T cell responses were analyzed by IFN-γ ELISPOT assays as previously described [19] with the following modifications: plates were coated with 8 µg rat anti-mouse IFN-γ MAb ml^−1^ (AN18; Mabtech AB, Stockholm, Sweden); DMEM was used; final cell concentration was 1×10^6^ cells/well; and for detection, horseradish peroxidase-conjugated strepavidin (Sigma-Aldrich, St Louis, MO) and DAB tablets (Sigma-Aldrich, St Louis, MO) were used. Spots were counted using an automated ELISPOT reader system ELR02 (Autoimmun Diagnostika GmbH, Strassberg, Germany).

The sequences of the ELISPOT peptides (Auspep and Mimotope, Melbourne, Australia), their definition as CD4 versus CD8 epitopes and use in ELISPOT, were originally described in Muller et al [19]. Peptides gD53, gD73, gD157 and gD273 correspond to amino acids 53–65, 73–85, 157–169, and 273–285, respectively, of gD2. Final concentration of DMSO in wells was 0.01%.

### Anti-gD1_21–339_ ELISA

ELISAs were performed as previously described [43]. Antibody titers for the unchallenged animals immunized as for the ELISPOT assay study were calculated as follows: splines were fitted to the dilution data in GraphPad Prism 5 and the inverse of the dilution required to give an OD of 0.5 was taken as the titer. A one-way ANOVA followed by Tukey’s Multiple Comparison test was used to determine if groups were significantly different.

### Anti-gD2 ELISA

Serum samples from the animals immunized but unchallenged were analyzed by ELISA using plates coated with C-terminally His-tagged gD2_26–331_ protein. A construct encoding C-terminally His-tagged gD2_1–331_ protein (pcDNA3-gD2tr-His) was made by PCR using pcDNA3-gD2 as a template. CHO cells were cultured in DMEM containing 10% fetal bovine serum (FBS; DKSH, Melbourne, Australia), penicillin, streptomycin and glutamine (Invitrogen, Carlsbad, CA) at 37°C and 5% CO_2_. The cells were transfected with empty vector or pcDNA3-gD2tr-His using Lipofectamine and Plus Reagent in OptiMEM (Invitrogen, Carlsbad, CA) according to the manufacturer’s recommendations. Supernatants were collected 40 hours after transfection and stored at −20°C. The protein in the supernatant is predicted to be C-terminally His-tagged gD2_26–331_ after the first 25 residues are cleaved from gD2_1–331-His tag_ when it is secreted from the cells.

Maxisorp microtiter plates (Nunc, Roskilde, Denmark) were coated overnight at 4°C with 50 µl of the gD2tr-His supernatant or control supernatant per well. After coating, plates were washed twice with PBS/0.1% Tween 20 (PBS-T) and then blocked for two hours at 37°C with 100 µl of 5% skim milk powder in PBS-T. After blocking, plates were washed with PBS-T and 50 µl of mouse sera at a dilution of 1 in 100 was added for one hour at 37°C. All serum was assayed in duplicate wells. Plates were washed again and 50 µl of sheep anti-mouse IgG horseradish peroxidase conjugate (Sigma, St. Louis, MO) was added at a 1 in 1000 dilution. After 1 hour, plates were washed and 100 µl of OPD (*o*-phenylenediamine dihydrochloride) substrate (Sigma, St. Louis, MO) was added. Absorbance was measured after 30 min and the addition of 25 µl of 3N HCl, at 492 nm in a Multiskan EX plate reader (Pathtech; Melbourne, Australia). Pre-immune sera and empty vector control sera all had OD492 values of less than 0.145 at a dilution of 1 in 100. Data was graphed and analyzed in GraphPad Prism 5 (GraphPad Inc.) by one-way ANOVA followed by Tukey’s Multiple Comparison test. P<0.05 was considered significant. The titers of groups which gave mean ODs greater than 1.0 were determined by repeating the ELISA using two-fold dilutions in 6 steps of the individual mouse serum samples in PBS i.e. from 1∶200 through to 1∶6400.

### Viral Challenge Studies

Six days prior to viral challenge, mice were treated with 2 mg of medroxyprogesterone (Depo-Provera, Pharmacia, Piscataway, NJ) subcutaneously [44]. Mice were anaesthetized with ketamine and xylazine mixture, and inoculated intravaginally with 1.55×10^4^ p.f.u. of HSV-2 strain 186, 50 times the dose required to kill 50% of age- and strain-matched animals. A 500 times dosage was also used. Virus was diluted in PBS. Vaginas were cleaned using sterile polyester urethral swabs (Copan, Murrieta, CA) and 10 µl of virus was injected into the vagina. Mice were monitored for 21 days. Animals showing signs of hind limb paralysis or other pre-morbid signs were euthanized. Positive control vaccination was performed using intravaginal inoculation 42 days prior to challenge with 5×10^5^ p.f.u./mouse thymidine kinase-deficient live HSV-2 strain 333 after progesterone treatment as previously described [19].

The second challenge study was carried out as for the first study except that the investigators were blinded, and mice were kept to day 102–106 and the lumbosacral DRG then dissected [45] for detection of HSV-2 DNA.

### HSV-2 Replication

HSV-2 replication was measured in all virus-challenged animals. Vaginal swabs were collected on days 1, 3 and 5 post-infection to determine HSV-2 DNA copy number. In addition, for the second study, animals that survived challenge were examined for viral replication in the DRG. 16 to 24 ganglia were carefully pooled per mouse into DNA extraction buffer. Mouse DNA genomes were measured with a real-time quantitative PCR primer/probe cocktail amplifying the glyceraldehyde-3-phosphate dehydrogenase gene (part 4308313, ABI, Foster City, CA). Methods used for DNA extraction and determination of copy number in the vaginal swabs and DRG have been previously described [32], [43], [46].

### Statistical Analysis

Statistical analysis was performed using GraphPad Prism version 5.03 for Windows, GraphPad Software, San Diego, CA, www.graphpad.com. Survival curves were compared by the log-rank (Mantel-Cox) test. HSV-2 peptide-specific T cell responses were compared by unpaired two-tailed t-tests and antibody titers by One-way ANOVA followed by Tukey’s Multiple Comparison test. HSV-2 titers in vaginal swabs were analyzed by calculating the area under the log_10_ titer-time curves (AUC) and comparing the AUCs using a Kruskal-Wallis test with Dunn’s multiple comparison. Differences were considered significant if P<0.05.

## Supporting Information

Figure S1 **Comparison of different vaccination regimes using NTC8485-O2-gD2 and NTC8485-O2-Ubi-gD2_25–331_ in the HSV-2 challenge model.** A. The effect of immunization on the survival of mice vaginally challenged with 50× LD_50_ (1.55×10^4^ p.f.u.) of live HSV-2 strain 186. +ve refers to the positive control mouse thymidine kinase-deficient live HSV-2 strain 333 (5×10^5^ p.f.u./mouse) vaccine; -ve refers to empty NTC8485. Survival rates of the active vaccine groups were all significantly higher than the negative control as determined by a log-rank (Mantel-Cox) test (P<0.05). The differences between active vaccine groups were not significant. B. Dorsal root ganglia HSV-2 DNA copy number at euthanasia in survivors of the 500× LD_50_ challenge 62±2 days after challenge. The ratios indicate the µg of non-ubiquitinated construct: µg of ubiquitinated construct; “x2” or “x3” indicate that the vaccine was administered twice or three times, respectively. The geometric means and 95% confidence intervals are shown. Ten mice/group/challenge dose were used (with the exception of the positive control which used five mice/challenge dose).
(TIF)

Figure S2 **HSV-2 copy number in swabs from mice immunized twice with NTC-based vaccines, taken after challenge.** Prior to intravaginal challenge with 50 (1.55×10^4^ p.f.u.) or 500×LD_50_ of live HSV-2 strain 186, mice were immunized with the indicated vaccines. 10 mice/group/challenge dose were used (with the exception of the positive control which used 5 mice/challenge dose). Vaginal swabs were taken 1, 3, and 5 days post-challenge. The ratios indicate the µg of non-ubiquitinated construct: µg of ubiquitinated construct; “x2” indicates that the vaccine was administered twice. The geometric means and 95% confidence intervals are shown.
(TIF)
