# Supplementary figures and images for: A Novel DNA Vaccine Technology Conveying Protection against a Lethal Herpes Simplex Viral Challenge in Mice
Source: PLoS One. 2013 Oct 3;8(10):e76407. doi: 10.1371/journal.pone.0076407 (PMC3789751; doi:10.1371/journal.pone.0076407)

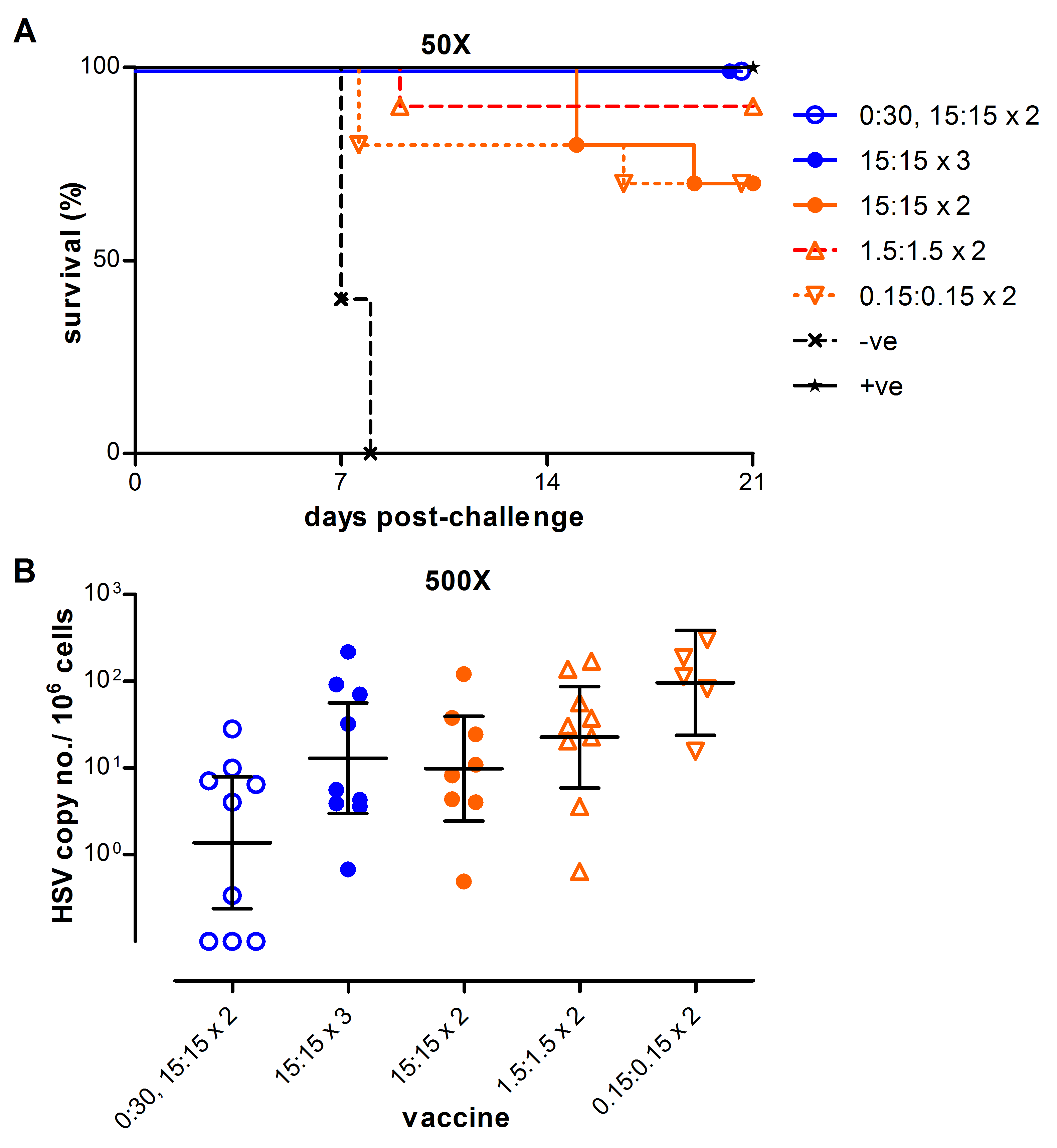

Supplement: Figure S1 — Comparison of different vaccination regimes using NTC8485-O2-gD2 and NTC8485-O2-Ubi-gD225–331 in the HSV-2 challenge model. A. The effect of immunization on the survival of mice vaginally challenged with 50× LD50 (1.55×10^4^ p.f.u.) of live HSV-2 strain 186. +ve refers to the positive control mouse thymidine kinase-deficient live HSV-2 strain 333 (5×10^5^ p.f.u./mouse) vaccine; -ve refers to empty NTC8485. Survival rates of the active vaccine groups were all significantly higher than the negative control as determined by a log-rank (Mantel-Cox) test (P<0.05). The differences between active vaccine groups were not significant. B. Dorsal root ganglia HSV-2 DNA copy number at euthanasia in survivors of the 500× LD50 challenge 62±2 days after challenge. The ratios indicate the µg of non-ubiquitinated construct: µg of ubiquitinated construct; “x2” or “x3” indicate that the vaccine was administered twice or three times, respectively. The geometric means and 95% confidence intervals are shown. Ten mice/group/challenge dose were used (with the exception of the positive control which used five mice/challenge dose). (TIF) [file pone.0076407.s001.tif]

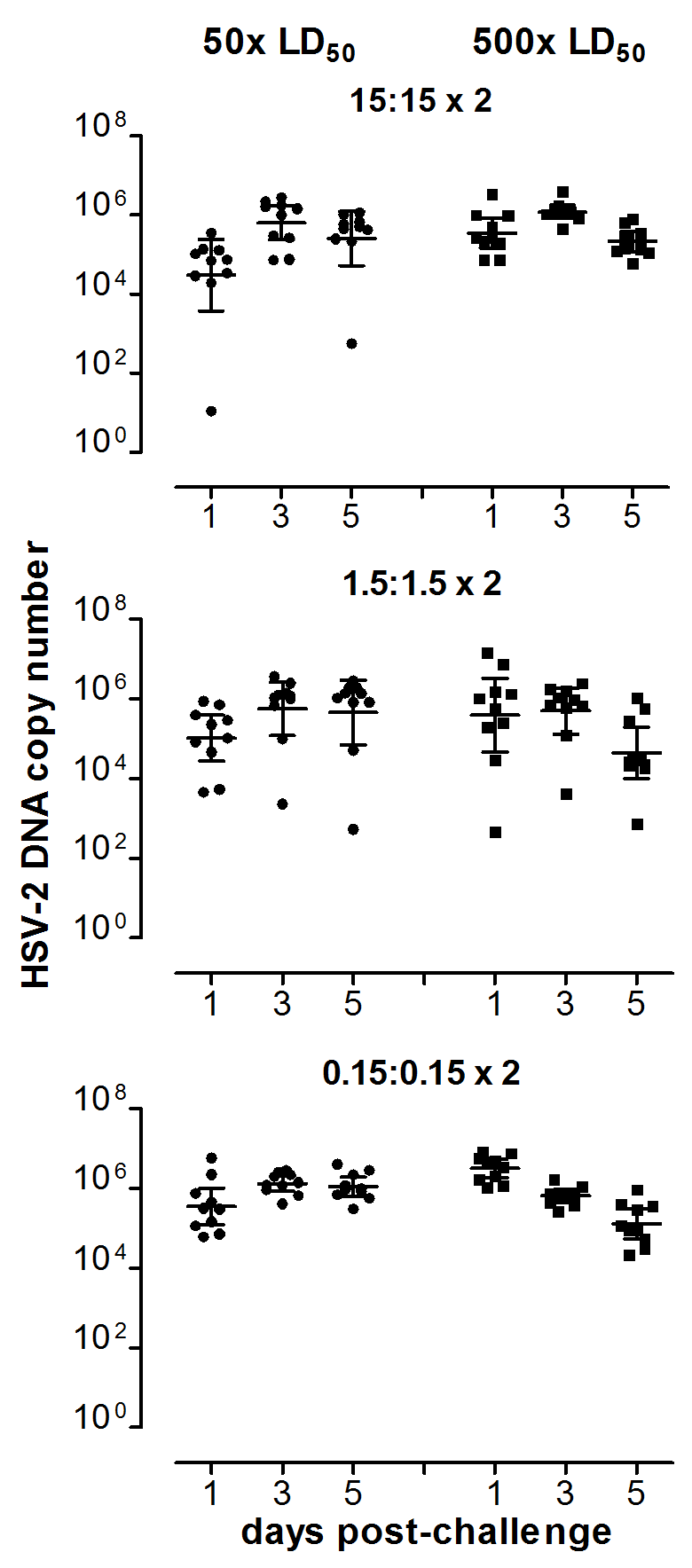

Supplement: Figure S2 — HSV-2 copy number in swabs from mice immunized twice with NTC-based vaccines, taken after challenge. Prior to intravaginal challenge with 50 (1.55×10^4^ p.f.u.) or 500×LD50 of live HSV-2 strain 186, mice were immunized with the indicated vaccines. 10 mice/group/challenge dose were used (with the exception of the positive control which used 5 mice/challenge dose). Vaginal swabs were taken 1, 3, and 5 days post-challenge. The ratios indicate the µg of non-ubiquitinated construct: µg of ubiquitinated construct; “x2” indicates that the vaccine was administered twice. The geometric means and 95% confidence intervals are shown. (TIF) [file pone.0076407.s002.tif]
